# Evaluation of Inflammatory, Immunonutritional, and Atherogenic Profiles in Patients with Hashimoto’s Thyroiditis

**DOI:** 10.3390/jcm15218607

**Published:** 2026-11-05

**Authors:** Güray Ceylan, Muammer Bilici, Ünzile Işıl Yüce

**Affiliations:** Department of Internal Medicine, Faculty of Medicine, Zonguldak Bulent Ecevit University, Zonguldak 67100, Turkey

**Keywords:** Hashimoto’s thyroiditis, nutrition, inflammation, systemic inflammation response index, atherogenic index of plasma, lipids

## Abstract

**Background/Objectives**: Hashimoto’s thyroiditis (HT) is associated with autoimmune thyroid inflammation, but its systemic inflammatory, immunonutritional, and atherogenic lipid profile across thyroid functional groups remains insufficiently characterized. This study aimed to evaluate routinely available inflammatory, immunonutritional, and atherogenic indices across the HT spectrum and to examine their relationships with thyroid function and autoimmunity. **Methods**: This retrospective study included newly diagnosed, untreated adult patients with HT and healthy controls without documented comorbidities. After exclusions and propensity score matching for age, sex, and body mass index, 148 patients with HT and 74 controls were analyzed. Patients with HT were classified as euthyroid HT (EH, *n* = 62), subclinical hypothyroidism (SCH, *n* = 47), or overt hypothyroidism (OH, *n* = 39). A total of 44 demographic, thyroid-related, hematologic, biochemical, lipid, inflammatory, immunonutritional, and atherogenic variables were evaluated. Elastic Net–penalized logistic regression, multivariable logistic regression, correlation analysis, and receiver operating characteristic curve analysis were performed. **Results**: HT was associated with a distinct systemic profile characterized by higher inflammatory and atherogenic indices and alterations in immunonutritional indices. Across the control, EH, SCH, and OH groups, systemic inflammation response index (SIRI; 0.5 vs. 0.8 vs. 1.0 vs. 1.4, *p* < 0.001) and atherogenic index of plasma (AIP; 0.1 vs. 0.3 vs. 0.5 vs. 0.8, *p* < 0.001) showed an ascending pattern, whereas prognostic nutritional index (PNI; 56.0 ± 3.2 vs. 50.2 ± 6.0 vs. 46.8 ± 5.5 vs. 40.6 ± 7.0, *p* < 0.001) showed a descending pattern. In multivariable models, SIRI and AIP were independently associated with EH compared with controls, whereas SIRI, AIP, and PNI were independently associated with SCH compared with EH and with OH compared with SCH. SIRI and AIP showed strong positive correlations with thyroid-stimulating hormone and negative correlations with free thyroxine, while PNI showed the opposite pattern. These indices also differed significantly according to anti-thyroid peroxidase and anti-thyroglobulin antibody status. **Conclusions**: Distinct inflammatory, immunonutritional, and atherogenic lipid profiles were observed across HT groups. SIRI and AIP may reflect systemic alterations even in EH, whereas lower PNI may reflect immunonutritional alterations associated with SCH or OH.

## 1. Introduction

Hashimoto’s thyroiditis (HT) is a chronic organ-specific autoimmune disease and the leading cause of chronic inflammation of the thyroid gland [1]. It affects an estimated 5–10% of the population and shows a marked female predominance [2]. Pathologically, HT is characterized by a dense lymphocytic infiltration of the thyroid gland and the production of anti-thyroid antibodies (notably anti-thyroid peroxidase and anti-thyroglobulin), which drive chronic inflammation, progressive fibrosis, and gradual destruction of thyroid follicles [3]. Over time, this autoimmune process leads to declining thyroid hormone levels and clinical hypothyroidism [4]. Importantly, the impact of HT is not confined to the thyroid gland: the ongoing autoimmune inflammation can have systemic consequences, creating a state of low-grade inflammation that may contribute to elevated cardiovascular risk in affected patients [5].

Systemic inflammation is intricately linked with thyroid function. On one hand, hypothyroidism is associated with a pro-inflammatory milieu, and on the other, certain inflammatory cytokines can inhibit thyroid hormone activity, suggesting a bidirectional interplay between the thyroid axis and immune activation [6]. Thyroid hormones themselves are key regulators of lipid metabolism; even subclinical thyroid dysfunction can lead to impaired lipid clearance and dyslipidemia, while restoring euthyroidism tends to improve serum lipid profiles [7]. Furthermore, there is a well-recognized bidirectional relationship between inflammation and lipid homeostasis: chronic inflammation can induce pro-atherogenic lipid changes such as increased triglyceride levels and reduced high-density lipoprotein cholesterol (HDL-C), and conversely, modified lipid species such as oxidized low-density lipoprotein (LDL) perpetuate inflammatory processes [8]. Experimental studies have also demonstrated a link between lipid metabolism and thyroid axis regulation. In animal models subjected to high-fat diets, alterations in thyroid function have been observed even in the absence of overt hormonal deficiency. Prolonged exposure to dietary fat has been associated with increased iodine uptake by the thyroid gland and elevated thyroid-stimulating hormone (TSH) levels, while circulating triiodothyronine (T3) and thyroxine (T4) concentrations remained within normal limits in the early stages [9]. In longer-term models, persistently elevated TSH levels have been observed together with reductions in total T4 and free thyroxine (fT4) [10]. These findings underscore the existence of a complex, reciprocal relationship between inflammation, lipid metabolism, and thyroid function. However, this interplay remains insufficiently explored, especially in human studies evaluating the combined impact of these processes across different stages of thyroid dysfunction.

To better capture the observed alterations in lipid and inflammatory profiles, several composite indices have been proposed. On the lipid side, the atherogenic index of plasma (AIP) reflects the combined effect of elevated triglycerides and reduced HDL-C and has been shown to correlate strongly with small dense LDL particles and increased cardiovascular risk [11]. Likewise, the Castelli Risk Index I (total cholesterol/HDL-C) and Castelli Risk Index II (low-density lipoprotein cholesterol [LDL-C]/HDL-C) summarize the balance between atherogenic and protective lipoproteins [12]. In parallel, complete blood count–derived indices such as the neutrophil-to-lymphocyte ratio (NLR), platelet-to-lymphocyte ratio (PLR), the systemic immune-inflammation index (SII), and the systemic inflammation response index (SIRI) have been introduced as inexpensive, integrative markers of systemic immune activation [13]. Several of these indices have been associated with HT and significantly correlated with thyroid function [14,15,16,17,18,19].

Despite the known links between thyroid function, inflammation, immunonutritional, and lipid metabolism, there is a notable lack of studies examining the intersection of systemic inflammation and atherogenic lipid indices specifically in HT. Prior research has typically addressed dyslipidemia in HT or systemic inflammatory markers in HT as separate issues, and the combined impact of HT-related inflammation on atherogenic lipid profiles remains unclear [14,15,16,17,18,19]. To address this gap, this study aimed to evaluate the levels of systemic inflammatory, immunonutritional, and atherogenic lipid indices across the spectrum of HT and to explore the relationships between these indices.

## 2. Materials and Methods

This retrospective study included adult patients with a diagnosis of HT who were followed in the Department of Internal Medicine at Zonguldak Bülent Ecevit University Faculty of Medicine between January 2015 and January 2025. The study protocol adhered to the ethical principles of the Declaration of Helsinki and was approved by the Zonguldak Bülent Ecevit University Faculty of Medicine Clinical Research Ethics Committee (approval date: 17 December 2025; decision no: 2025/22). Due to the retrospective nature of the study, the requirement for informed consent was waived by the Ethics Committee.

### 2.1. Study Design and Population

Due to the retrospective nature of the study, a traditional prospective sample size calculation was not performed prior to data collection. Instead, the sample was based on available clinical records, from which 248 patients with HT aged over 18 years were screened for eligibility during the study period. The inclusion criteria comprised newly diagnosed patients with HT who had no additional comorbidities and had not yet initiated any form of medical treatment. Exclusion criteria included any previously documented any additional disease, such as diabetes mellitus, hypertension, arterial disease, chronic kidney disease, or malignancy; use of antioxidant agents, lipid-lowering medications, or vitamin supplements; and missing data. Additionally, individuals who smoked or consumed any amount of alcohol were not included in the study. After applying these exclusion criteria, 176 patients with HT were deemed eligible for inclusion.

For the control group, medical records of 120 healthy individuals over the age of 18 who presented to the hospital for routine check-ups and had no documented comorbidities were reviewed. To address the imbalance in covariates [age, sex, and body mass index (BMI)] between the HT patients and control groups, propensity score matching analysis was performed. The analysis involved 2:1 matching with the nearest neighbor matching method, using calipers (0.2) with a width equal to 0.25 of the standard deviation of the logit [20]. Individuals who did not have a match were excluded from the study. As a result, the analysis included 148 patients with HT and 74 healthy individuals in the control group.

### 2.2. Data Collection and Definitions

Demographic, clinical, and laboratory data were retrieved retrospectively from the hospital’s electronic medical records and archived patient files. All laboratory values used in the study were obtained at the time of initial diagnosis and reflected the patients’ baseline clinical status. As per institutional practice, fasting venous blood samples are routinely collected in the morning, and the values analyzed in this study were derived from previously recorded test results. The continuity of analytical platforms and assay methods throughout the 2015–2025 study period could not be retrospectively verified. Accordingly, measurement comparability across the full study period and cross-platform harmonization could not be confirmed. Details of the collected dataset are summarized in Supplementary Table S1. Briefly, these variables were grouped under demographic data, thyroid function markers, hematological parameters, acute-phase reactants, lipid profiles, and derived inflammatory, immunonutritional, and atherogenic indices.

HT was identified based on the presence of positive serum anti-thyroglobulin (anti-Tg) and/or anti-thyroid peroxidase (anti-TPO) antibodies, or by the detection of heterogeneous echogenicity in the thyroid parenchyma on ultrasonographic examination. Following diagnosis, patients were classified according to their thyroid function test results: those with both TSH and fT4 levels within reference ranges were classified as having euthyroid HT (EH); individuals with elevated TSH but normal fT4 levels were categorized as having subclinical hypothyroidism (SCH); and those with increased TSH accompanied by reduced fT4 were classified as having overt hypothyroid-ism (OH).

All individuals in the control group exhibited normal thyroid function, as evidenced by serum TSH, fT4, anti-Tg, and anti-TPO levels within reference ranges. In addition, thyroid ultrasonography revealed a homogeneous glandular echotexture without nodular formations in all control subjects.

BMI was calculated as weight in kilograms divided by height in meters squared (kg/m^2^).

### 2.3. Inflammatory, Immunonutritional, and Atherogenic Indices

Composite inflammatory, immunonutritional, and atherogenic indices were calculated using routinely measured hematological and biochemical parameters (Supplementary Table S2). Briefly, inflammatory indices included NLR, PLR, the lymphocyte-to-monocyte ratio (LMR), SII, SIRI, and the C-reactive protein (CRP)-to-albumin ratio (CAR). The prognostic nutritional index (PNI) was calculated according to the Onodera formula using serum albumin and total lymphocyte count [21]. To assess atherogenic lipid status, the following indices were computed from fasting lipid panel results: AIP was calculated according to the definition proposed by Dobiášová and Frohlich as the base-10 logarithm of the molar triglyceride-to-HDL-C ratio [22]. Accordingly, triglyceride and HDL-C concentrations reported in mg/dL were converted to mmol/L before calculating AIP. The Castelli Risk Indices I and II were calculated as total cholesterol/HDL-C and LDL-C/HDL-C, respectively.

### 2.4. Statistical Analysis

All statistical procedures were conducted using the Statistical Package for the Social Sciences (SPSS) version 24.0 (IBM Corp., Armonk, NY, USA) and Python version 3.13.5 (Python Software Foundation, Wilmington, DE, USA). The distribution of continuous variables was assessed using the Kolmogorov–Smirnov test. Normally distributed variables were expressed as mean ± standard deviation, whereas non-normally distributed variables were presented as median with the interquartile range. Categorical variables were summarized as numbers and percentages. For two-group comparisons, Student’s *t*-test or the Mann–Whitney U test was used according to distributional normality. For comparisons among more than two groups, one-way analysis of variance (ANOVA) or the Kruskal–Wallis test was applied, with Bonferroni or Dunn–Bonferroni post-hoc correction when appropriate. Categorical variables were compared using the chi-square test or Fisher’s exact test.

Pearson or Spearman correlation analyses were applied to evaluate the relationships between numerical variables. Correlation strength was classified according to coefficient magnitude: <0.10, negligible; 0.10–0.39, weak; 0.40–0.69, moderate; 0.70–0.89, strong; and 0.90–1.00, very strong [23]. Correlations between thyroid-related parameters (anti-Tg, anti-TPO, TSH, and fT4) and inflammatory, immunonutritional, and atherogenic indices were evaluated. Additional within-group correlation analyses were performed to examine the associations of PNI and Controlling Nutritional Status (CONUT) score with inflammatory and atherogenic indices. For the full correlation panel, multiple testing was additionally controlled using the Benjamini–Hochberg false discovery rate (FDR) procedure, and FDR-adjusted q values were reported.

Because several candidate variables were mathematically or biologically correlated, Elastic Net–penalized logistic regression was used for variable reduction and selection [24]. Separate Elastic Net models were constructed for HT versus controls and for the predefined pairwise group comparisons: EH versus controls, SCH versus EH, and OH versus SCH. Candidate variables included demographic and clinical parameters together with inflammatory, immunonutritional, and atherogenic indices. Thyroid-defining variables, including TSH, fT4, anti-TPO, and anti-Tg, were not entered into the Elastic Net models to avoid circular classification. Continuous candidate predictors were standardized to a mean of 0 and a standard deviation of 1 before penalized modeling to ensure comparable penalization across variables. The Elastic Net mixing parameter, implemented as the l1_ratio, was evaluated over a prespecified grid of 0.10, 0.25, 0.50, 0.75, and 0.90. For each l1_ratio value, the inverse regularization-strength parameter (C) was optimized across a log10(C) grid using 10-fold stratified cross-validation, with the area under the receiver operating characteristic (ROC) curve (AUC) as the performance criterion. The l1_ratio–C combination yielding the highest mean cross-validated AUC was selected separately for each model. Variables retaining non-zero coefficients at the selected regularization level were considered informative variables. Elastic Net models were fitted using the LogisticRegression implementation with the SAGA solver in scikit-learn 1.8.0 (Python 3.13.5).

Parameters identified as important by Elastic Net models were subsequently entered into multivariable logistic regression models to determine independent predictors. Results were expressed as odds ratios (ORs) with corresponding 95% confidence intervals (CIs). ROC curve analyses were then used to assess the diagnostic performance of laboratory parameters and composite indices. Optimal cutoff values were derived using the Youden index, and differences between AUCs of independent predictors were compared with the DeLong test [25]. A two-tailed *p*-value < 0.05 was considered statistically significant.

## 3. Results

### 3.1. Study Population

A total of 222 participants were included in the study, comprising 74 controls and 148 patients with HT. Among the HT patients, 62 were EH, 47 had SCH, and 39 had OH. In the overall population, mean age was 43.1 ± 14.3 years, mean BMI was 27.5 ± 5.1 kg/m^2^, and 74.3% of participants were female. Among patients with HT, median TSH was 6.0 (IQR, 2.8–7.5) mIU/L and median fT4 was 0.8 (IQR, 0.6–1.0) ng/dL, while anti-TPO and anti-Tg positivity were observed in 84.5% and 62.2% of patients, respectively. Patients with HT had a higher BMI than controls (28.3 ± 5.4 vs. 26.5 ± 4.4 kg/m^2^, *p* = 0.033). In addition, alterations were observed in total leukocyte and differential counts as well as in lipid profile parameters, while serum albumin levels were lower in the HT group. Detailed demographic and laboratory characteristics of study population are provided in Supplementary Table S3.

Inflammatory indices were higher in patients with HT than in controls, including NLR (2.0 vs. 1.3, *p* < 0.001), PLR (120.7 vs. 101.3, *p* = 0.016), SII (516.7 vs. 306.6, *p* < 0.001), SIRI (1.0 vs. 0.5, *p* < 0.001), and CAR (0.8 vs. 0.5, *p* < 0.001). LMR was lower in patients with HT (4.1 vs. 5.6, *p* < 0.001). Regarding immunonutritional indices, patients with HT had lower PNI (46.1 ± 8.0 vs. 56.0 ± 3.2, *p* < 0.001) and higher CONUT scores (1.0 vs. 0.0, *p* < 0.001). Atherogenic indices were also higher in the HT group, including AIP (0.5 vs. 0.1, *p* < 0.001), Castelli I index (4.1 ± 1.0 vs. 3.3 ± 0.9, *p* < 0.001), and Castelli II index (2.5 ± 0.8 vs. 2.0 ± 0.7, *p* < 0.001) (Table 1).

### 3.2. Associations Between Thyroid Markers and Systemic Indices

TSH showed positive correlations with NLR (ρ = 0.502, *p* < 0.001), SII (ρ = 0.425, *p* < 0.001), SIRI (ρ = 0.668, *p* < 0.001), CAR (ρ = 0.432, *p* < 0.001), CONUT score (ρ = 0.348, *p* < 0.001), AIP (ρ = 0.649, *p* < 0.001), Castelli I index (ρ = 0.487, *p* < 0.001), and Castelli II index (ρ = 0.366, *p* < 0.001), whereas it was negatively correlated with LMR (ρ = −0.392, *p* < 0.001) and PNI (ρ = −0.548, *p* < 0.001). No significant correlation was observed between TSH and PLR (ρ = 0.122, *p* = 0.071).

fT4 was negatively correlated with NLR (ρ = −0.442, *p* < 0.001), SII (ρ = −0.333, *p* < 0.001), SIRI (ρ = −0.515, *p* < 0.001), CAR (ρ = −0.190, *p* = 0.004), CONUT score (ρ = −0.381, *p* < 0.001), AIP (ρ = −0.406, *p* < 0.001), Castelli I index (ρ = −0.305, *p* < 0.001), and Castelli II index (ρ = −0.218, *p* = 0.001), while positive correlations were observed with LMR (ρ = 0.310, *p* < 0.001) and PNI (ρ = 0.452, *p* < 0.001). PLR was not significantly correlated with fT4 (ρ = −0.074, *p* = 0.270).

Anti-TPO positivity was positively correlated with NLR (ρ = 0.394, *p* < 0.001), SII (ρ = 0.418, *p* < 0.001), SIRI (ρ = 0.529, *p* < 0.001), CAR (ρ = 0.167, *p* = 0.013), CONUT score (ρ = 0.194, *p* = 0.004), AIP (ρ = 0.516, *p* < 0.001), Castelli I index (ρ = 0.345, *p* < 0.001), and Castelli II index (ρ = 0.278, *p* < 0.001), and negatively correlated with LMR (ρ = −0.215, *p* = 0.001) and PNI (ρ = −0.455, *p* < 0.001). Its correlation with PLR was not significant (ρ = 0.087, *p* = 0.196).

Similarly, anti-Tg positivity showed positive correlations with NLR (ρ = 0.411, *p* < 0.001), SII (ρ = 0.346, *p* < 0.001), SIRI (ρ = 0.464, *p* < 0.001), CAR (ρ = 0.171, *p* = 0.011), CONUT score (ρ = 0.157, *p* = 0.019), AIP (ρ = 0.364, *p* < 0.001), Castelli I index (ρ = 0.262, *p* < 0.001), and Castelli II index (ρ = 0.234, *p* < 0.001), whereas negative correlations were observed with LMR (ρ = −0.195, *p* = 0.004) and PNI (ρ = −0.360, *p* < 0.001). No significant correlation was observed with PLR (ρ = 0.091, *p* = 0.176).

**Figure 1:**
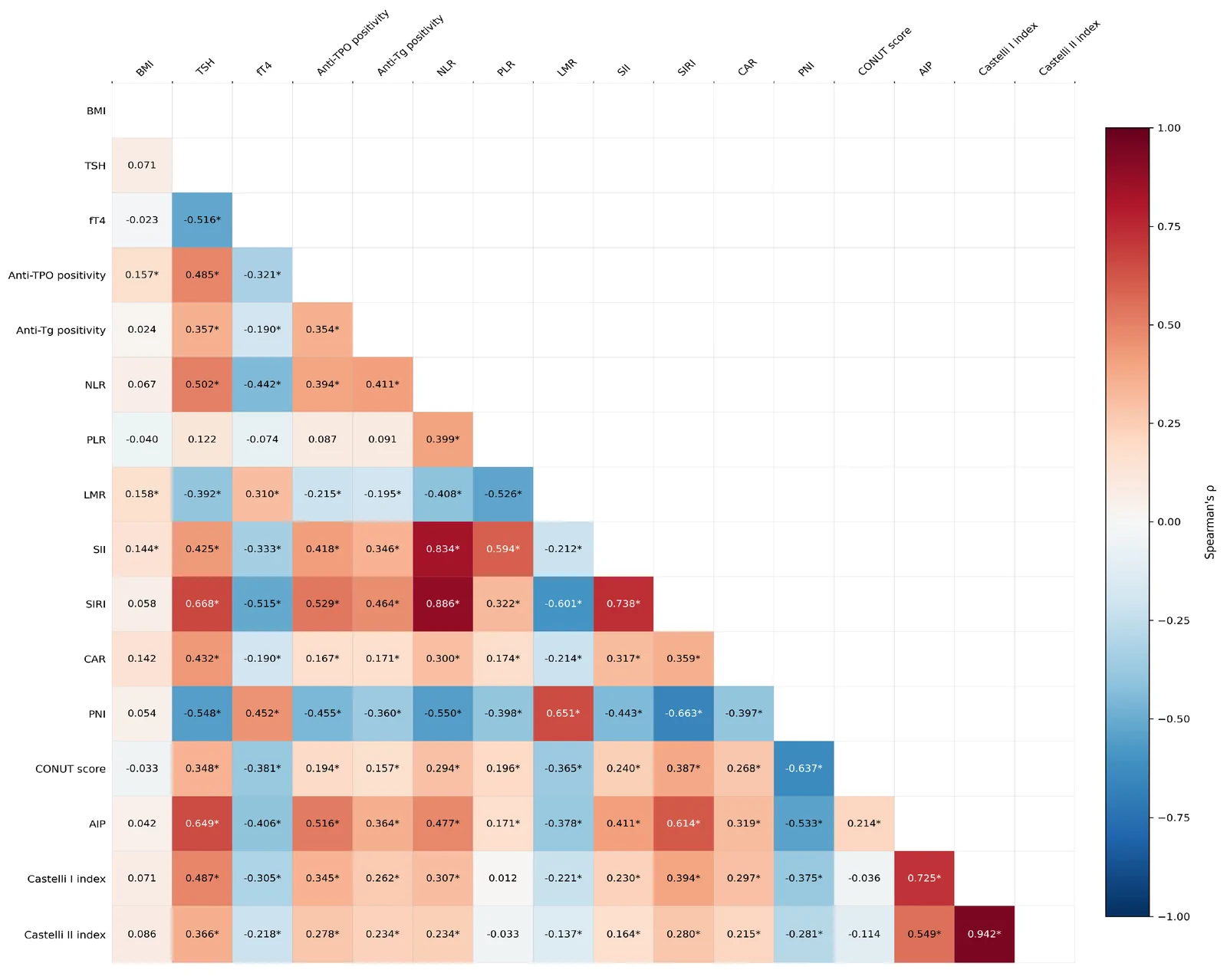
Spearman correlation heatmap of thyroid markers and inflammatory, immunonutritional, and atherogenic indices. Values within cells represent Spearman’s correlation coefficients (ρ). Red shades indicate positive correlations, whereas blue shades indicate negative correlations. Increasing color intensity reflects greater correlation strength, while lighter shades indicate weaker correlations. Asterisks (*) denote statistically significant correlations (*p* < 0.05). BMI, body mass index; TSH, thyroid-stimulating hormone; fT4, free thyroxine; anti-TPO, anti-thyroid peroxidase antibody; anti-Tg, anti-thyroglobulin antibody; NLR, neutrophil-to-lymphocyte ratio; PLR, platelet-to-lymphocyte ratio; LMR, lymphocyte-to-monocyte ratio; SII, systemic immune-inflammation index; SIRI, systemic inflammation response index; CAR, C-reactive protein-to-albumin ratio; PNI, prognostic nutritional index; CONUT, Controlling Nutritional Status; AIP, atherogenic index of plasma.

Correlations between thyroid markers and inflammatory, immunonutritional, and atherogenic indices are summarized in the heatmap presented in Figure 1.

Benjamini–Hochberg FDR correction did not change the significance pattern of the full correlation panel; corresponding Spearman correlation coefficients and FDR-adjusted q values are provided in Supplementary Table S4.

### 3.3. Inflammatory, Immunonutritional, and Atherogenic Profiles Across Thyroid Functional States

BMI was higher in OH than in the other HT subgroups (Control: 26.5 ± 4.4 vs. EH: 27.2 ± 5.6 vs. SCH: 28.0 ± 5.1 vs. OH: 30.3 ± 5.2 kg/m^2^, *p* = 0.038), whereas age and sex distributions were comparable. As expected from the classification criteria, TSH and fT4 values differed across thyroid functional groups, while anti-TPO and anti-Tg positivity differed markedly between controls and HT subgroups (all *p* < 0.001). Leukocyte, neutrophil, and monocyte counts showed an ascending pattern across thyroid functional groups, whereas albumin levels showed a descending pattern, with the lowest values observed in OH (all *p* < 0.001). Significant between-group differences were also observed in CRP and lipid parameters, with higher triglyceride, total cholesterol, and LDL-C levels and lower HDL-C levels in groups with more pronounced thyroid dysfunction. Demographic and clinical characteristics according to HT subgroup are presented in Supplementary Table S5.

Inflammatory indices differed significantly according to thyroid functional status. NLR was higher across HT subgroups, with the highest value observed in OH (Control: 1.2 vs. EH: 1.7 vs. SCH: 2.1 vs. OH: 2.6, *p* < 0.001). PLR also differed among the groups (Control: 101.3 vs. EH: 118.3 vs. SCH: 127.4 vs. OH: 136.4, *p* = 0.035), whereas LMR showed an inverse pattern (Control: 5.6 vs. EH: 4.7 vs. SCH: 4.3 vs. OH: 3.3, *p* < 0.001). CAR was similar between controls and EH but was higher in SCH and OH (Control: 0.5 vs. EH: 0.5 vs. SCH: 1.0 vs. OH: 1.3, *p* < 0.001). Across the control, EH, SCH, and OH groups, SII (Control: 306.6 vs. EH: 401.0 vs. SCH: 445.2 vs. OH: 504.0, *p* < 0.001) and SIRI (Control: 0.5 vs. EH: 0.8 vs. SCH: 1.0 vs. 1.4, *p* < 0.001) showed an ascending pattern, whereas PNI (Control: 56.0 ± 3.2 vs. EH: 50.2 ± 6.0 vs. SCH: 46.8 ± 5.5 vs. OH: 40.6 ± 7.0, *p* < 0.001) showed a descending pattern. The CONUT score was markedly higher in OH (Control: 0.0 vs. EH: 1.0 vs. SCH: 1.0 vs. OH: 3.0, *p* < 0.001). AIP showed an ascending pattern across the control, EH, SCH, and OH groups (Control: 0.1 vs. EH: 0.3 vs. SCH: 0.5 vs. OH: 0.8, *p* < 0.001). Similar patterns were observed for the Castelli I index (Control: 3.3 ± 0.9 vs. EH: 3.5 ± 0.7 vs. SCH: 4.3 ± 1.0 vs. OH: 4.9 ± 1.1, *p* < 0.001) and Castelli II index (Control: 2.0 ± 0.7 vs. EH: 2.2 ± 0.6 vs. SCH: 2.6 ± 0.8 vs. OH: 2.9 ± 0.9, *p* < 0.001) (Table 2).

### 3.4. Factors Associated with Thyroid Functional Status

Elastic Net–penalized logistic regression was performed to identify non-thyroid systemic markers associated with HT subgroups. Thyroid-defining variables, including TSH, fT4, anti-TPO, and anti-Tg, were not entered into the Elastic Net models.

**Figure 2:**
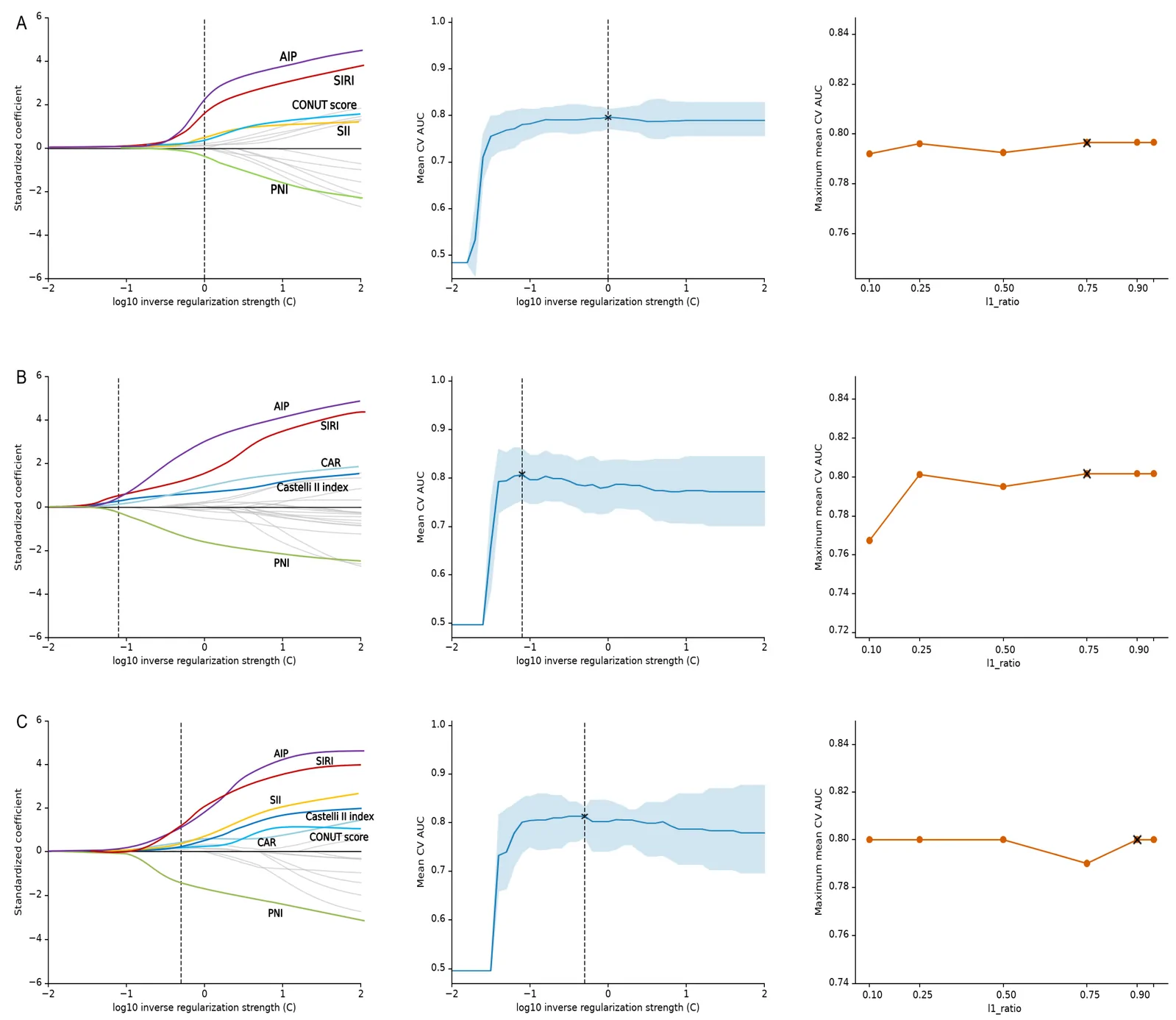
Elastic Net–based selection of systemic markers across Hashimoto’s thyroiditis (HT) subgroup. Elastic Net–penalized logistic regression was performed for the predefined pairwise comparisons: euthyroid HT versus controls (**A**), subclinical hypothyroidism versus euthyroid HT (**B**), and overt hypothyroidism versus subclinical hypothyroidism (**C**). Left panels show standardized coefficient paths across the log10 inverse regularization strength (**C**); colored lines indicate informative variables retained at the selected regularization level, whereas gray lines indicate variables not retained as informative. Middle panels show the area under the receiver operating characteristic curve (AUC), estimated using 10-fold cross-validation (CV), for the selected l1_ratio across C values, with the dashed vertical line indicating the selected regularization level. Right panels show l1_ratio selection based on the maximum mean cross-validated AUC across C values. Thyroid-defining variables, including TSH, fT4, anti-TPO, and anti-Tg, were not included in the Elastic Net models. The × symbol indicates the selected l1_ratio value. In the middle panels, blue lines represent the mean cross-validated AUC, and light-blue shaded areas indicate ±1 standard deviation (SD). Other abbreviations: see Figure 1.

For EH versus controls, the selected l1_ratio was 0.75, the selected log10 inverse regularization strength was 0.00, and the 10-fold cross-validated AUC was 0.794. The selected variables were SII, SIRI, PNI, CONUT score, and AIP (Figure 2A). For SCH versus EH, the selected l1_ratio was 0.75, the selected log10 inverse regularization strength was −1.10, and the 10-fold cross-validated AUC was 0.804. The selected variables were SIRI, CAR, PNI, AIP, and Castelli II index (Figure 2B). For OH versus SCH, the selected l1_ratio was 0.90, the selected log10 inverse regularization strength was −0.30, and the 10-fold cross-validated AUC was 0.802. The selected variables were SII, SIRI, CAR, PNI, CONUT score, AIP, and Castelli II index (Figure 2C).

Variables retained by the Elastic Net models were subsequently evaluated using conventional univariable and multivariable logistic regression analyses (Table 3). In the multivariable model, SIRI (OR = 2.15, 95% CI: 1.42–3.27, *p* < 0.001) and AIP (OR = 2.47, 95% CI: 1.62–3.77, *p* < 0.001) remained independently associated with EH, whereas SII, PNI, and CONUT score were no longer significant. Similarly, SIRI (OR = 1.75, 95% CI: 1.30–2.35, *p* < 0.001), PNI (OR = 0.92, 95% CI: 0.86–0.98, *p* < 0.001), and AIP (OR = 2.32, 95% CI: 1.46–3.70, *p* < 0.001) remained independently associated with SCH, whereas CAR and the Castelli II index lost statistical significance. Finally, SIRI (OR = 1.33, 95% CI: 1.03–1.72, *p* = 0.005), PNI (OR = 0.82, 95% CI: 0.70–0.96, *p* = 0.007), and AIP (OR = 2.15, 95% CI: 1.44–3.21, *p* = 0.001) remained independently associated with OH, while SII, CAR, CONUT score, and the Castelli II index were not significant.

### 3.5. Diagnostic Performance of Inflammatory, Immunonutritional, and Atherogenic Indices Across Thyroid Functional Groups

**Figure 3:**
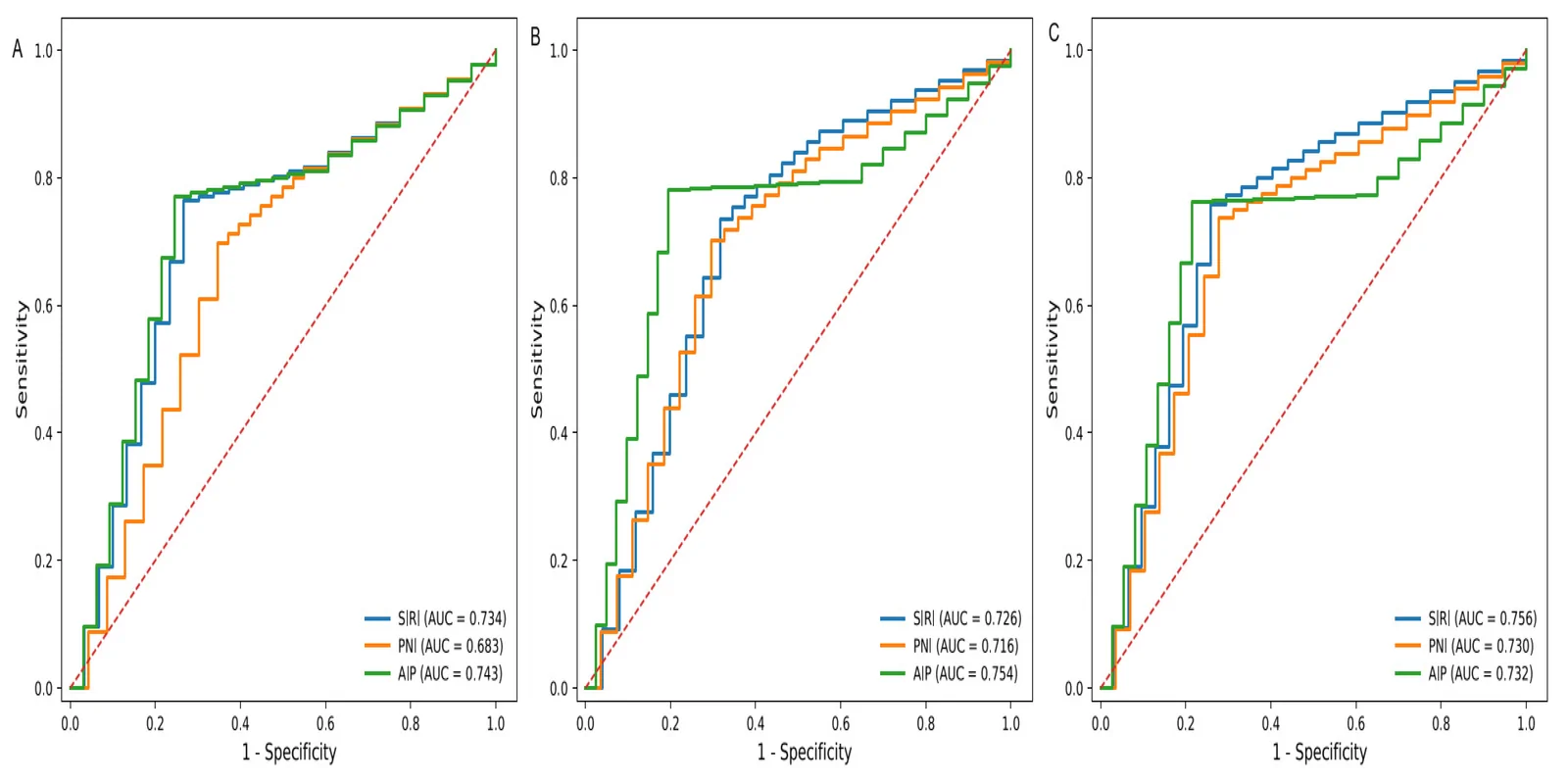
Diagnostic performance of the systemic inflammation response index (SIRI), prognostic nutritional index (PNI), and atherogenic index of plasma (AIP) in pairwise comparisons of thyroid functional groups: (**A**) euthyroid Hashimoto’s thyroiditis (EH) versus controls, (**B**) subclinical hypothyroidism (SCH) versus EH, and (**C**) overt hypothyroidism (OH) versus SCH.

ROC analysis showed that SIRI, PNI, and AIP significantly discriminated between the thyroid functional groups in the predefined pairwise comparisons (all *p* < 0.001; Supplementary Table S6 and Figure 3). For EH versus controls, AIP showed the highest numerical discrimination (AUC = 0.743 ± 0.04; cutoff ≥ 0.19; sensitivity 77.1%; specificity 75.5%), followed by SIRI (AUC = 0.734 ± 0.04) and PNI (AUC = 0.683 ± 0.04). AIP also had the largest AUC for distinguishing SCH from EH (AUC = 0.754 ± 0.04; cutoff ≥ 0.49; sensitivity 78.1%; specificity 80.5%), whereas the AUCs of SIRI and PNI were 0.726 ± 0.05 and 0.716 ± 0.05, respectively. For OH versus SCH, SIRI provided the highest numerical AUC (0.756 ± 0.06; cut-off ≥ 1.25; sensitivity 75.8%; specificity 74.2%), while AIP and PNI showed comparable performance (AUC = 0.732 ± 0.06 and 0.730 ± 0.04, respectively).

## 4. Discussion

This study comprehensively characterized the systemic inflammatory, immunonutritional, and atherogenic profiles of patients with HT across thyroid functional groups. By analyzing 44 routinely available laboratory-derived parameters in a propensity score–matched cohort, we identified a consistent systemic pattern across thyroid functional groups. Among the evaluated indices, SIRI, PNI, and AIP emerged as the most clinically informative markers. SIRI and AIP were independently associated with EH compared with healthy controls, suggesting that systemic inflammatory and atherogenic changes may be present even in the absence of overt biochemical hypothyroidism. In pairwise group comparisons, SIRI, AIP, and PNI were independently associated with SCH compared with EH and with OH compared with SCH. These findings suggest that different thyroid functional states are reflected not only in changes in TSH and fT4 but also in a broader systemic profile characterized by myeloid-predominant inflammation, alterations in immunonutritional indices, and a more atherogenic lipid profile.

Several previous studies have investigated complete blood count–derived inflammatory indices in HT, although most have focused on NLR, PLR, or SII rather than SIRI. Bilge et al. reported higher NLR and PLR in EH than in healthy controls, indicating that systemic inflammation may be present despite preserved thyroid hormone levels [26]. Chen et al. demonstrated that NLR and PLR were positively correlated with TSH and inversely correlated with free triiodothyronine (fT3) and FT4, supporting a close relationship between an increasingly unfavorable thyroid hormone profile and systemic inflammatory activity [27]. Similarly, Ghosh et al., in a cohort comprising EH controls and patients with SCH and OH, demonstrated progressively greater inflammatory activation with worsening thyroid hormone deficiency, with circulating pro-inflammatory markers and leukocyte inflammatory signaling being most pronounced in OH [28]. More recently, Hu et al. and Atak Tel et al. reported associations between SII and thyroid function or HT status [16,29]. whereas Zhai et al., using National Health and Nutrition Examination Survey (NHANES) data, demonstrated independent associations between SIRI and thyroid function parameters and TPOAb [15]. Consistent with these observations, SIRI showed the strongest positive correlation with TSH and a moderate negative correlation with fT4 in the present cohort, whereas the corresponding correlations for SII were weaker. SIRI was also markedly higher in both anti-TPO-positive and anti-Tg-positive patients. Together, these findings suggest that composite inflammatory indices, particularly SIRI, may reflect systemic inflammatory differences associated with thyroid functional status and thyroid autoimmunity.

To our knowledge, evidence from adult studies characterizing an ascending SIRI pattern across EH, SCH, and OH, together with its independent associations in predefined pairwise group comparisons, remains limited. The persistence of SIRI, but not SII, in the multivariable models may partly reflect differences in their cellular composition. Although both indices incorporate neutrophil and lymphocyte counts, SII additionally includes platelets, whereas SIRI incorporates monocytes. In the present cohort, platelet counts showed little variation across thyroid functional subgroups, while neutrophil and monocyte counts were higher in groups with more pronounced thyroid dysfunction. This pattern is biologically plausible given the immune architecture of HT: single-cell analyses by Zhang et al. demonstrated contributions of inflammatory macrophages, dendritic cells, and other immune-cell populations to lymphocyte trafficking and thyrocyte injury [30], while Bozdağ et al. similarly reported differences in neutrophil, lymphocyte, and monocyte counts across the HT spectrum without significant variation in platelet counts [31]. Although peripheral SIRI cannot directly quantify thyroidal immune infiltration, its consistent association with HT groups may therefore reflect a circulating counterpart of broader myeloid–lymphoid inflammatory activity. Complementary imaging evidence supports increased local thyroid metabolic activity in HT. Małkowski et al. evaluated 20 untreated patients with HT using fluorine-18 fluorodeoxyglucose (^18^F-FDG) positron emission tomography/computed to-mography (PET/CT) and reported diffuse thyroid uptake with higher maximum standardized uptake values (SUVmax) than in six controls in cancer remission without thyroid disease [32]. Edo et al., in a retrospective study of 18 patients with HT, also reported positive correlations between thyroid SUVmax and anti-TPO and anti-Tg titers [33].

Nutritional factors may themselves interact with the inflammatory milieu and thyroid function in HT. Although dietary intake and cytokine levels were not assessed in the present study, existing nutritional evidence provides indirect biological support for the observed inflammatory–nutritional–atherogenic pattern. In a randomized trial of patients with HT, Nigella sativa supplementation was associated with changes in thyroid function and autoimmunity, together with a reduction in interleukin (IL)-23, suggesting that nutritional or phytochemical interventions may influence both thyroid-axis parameters and cytokine-related immune activity [34]. More recently, dietary fiber intervention studies in HT have also evaluated thyroid function tests together with inflammatory cytokines such as IL-6 and tumor necrosis factor-α (TNF-α), supporting a potential gut–immune–thyroid interaction [35]. Experimental high-fat diet models further show that dietary metabolic stress can alter thyroid hormone profiles and increase inflammatory mediators [10,36]. Chen et al. reported that a more pro-inflammatory dietary pattern, reflected by a higher Dietary Inflammatory Index, was associated with higher TSH and total T4 levels in patients with HT [37].

Against this background, laboratory-based immunonutritional indices may complement inflammatory and atherogenic markers in characterizing the systemic profile of HT. PNI and CONUT both incorporate serum albumin and lymphocyte count, while CONUT additionally incorporates total cholesterol. Neither index remained independently associated with EH compared with controls after multivariable adjustment. Direct evidence regarding PNI or CONUT in HT remains limited. Önal et al. recently evaluated immunonutritional indices, inflammatory markers, and thyroid-related parameters in adults with HT and showed that PNI, CONUT score, and complete blood count–derived inflammatory markers were related to thyroid-related and metabolic parameters [38]. PNI is based on albumin and lymphocyte count, and its variation may reflect inflammatory activity and thyroid-related metabolic changes. Accordingly, lower PNI values should be interpreted as alterations in a laboratory-based immunonutritional profile rather than evidence of clinically established nutritional impairment. The cholesterol component of CONUT may additionally reflect hypothyroidism-related lipid alterations, complicating its interpretation in this setting. This may partly explain why PNI remained independently associated with SCH and OH, whereas CONUT lost significance after adjustment for AIP and inflammatory indices.

Chronic inflammation is known to alter lipid profiles. Santi et al. reported that patients with OH had elevated circulating levels of pro-inflammatory cytokines, including IL-1, IL-6, TNF-α, and interferon-γ (IFN-γ), which were significantly associated with lipid abnormalities [39]. This is consistent with the positive correlation observed between AIP and SIRI in patients with HT. Moreover, AIP showed a strong positive correlation with TSH and a moderate negative correlation with fT4, and it was higher in both anti-TPO-positive and anti-Tg-positive patients. The observation that AIP was independently associated with EH is particularly important. In a large retrospective study of 495 EH patients and 505 controls, Özkan and Harı reported higher AIP in EH, and AIP correlated with anti-TPO and anti-Tg levels [40]. This is consistent with the present finding that AIP discriminated EH from healthy controls and remained independently associated in the EH group. Li et al., using NHANES data, demonstrated a relationship between AIP and thyroid function, indicating that AIP may be linked to thyroid-axis variation at a population level [17]. In hypothyroid patients, Demirhan et al. also reported that AIP was related to TSH levels and thyroid autoantibodies [41]. Cengiz et al. showed that dyslipidemia may be evident even during the EH phase of HT, with significantly higher LDL-C, triglyceride, total cholesterol, and HDL-C levels in EH patients than in antibody-negative controls. Notably, anti-TPO and anti-Tg titers were positively correlated with LDL-C, triglycerides, and total cholesterol, suggesting that thyroid autoimmunity itself may contribute to adverse lipid alterations independently of overt thyroid dysfunction [42].

Studies conducted across different geographic regions have reported higher AIP levels in patients with SCH [18,19]. Chen et al. recently combined NHANES analysis with animal experimentation and reported an association between AIP and SCH, with TSH playing a mediating role in this relationship [43]. Couto et al. also described a complex interrelationship between thyroid function, autoimmunity, thyroid volume, and lipid profile in HT, highlighting that metabolic risk may not be restricted to OH [44]. These findings align with the between-group differences in AIP observed in our cohort and its independent associations in the predefined thyroid functional group comparisons. The mechanisms underlying this relationship are likely multifactorial. Thyroid hormones regulate hepatic cholesterol synthesis, LDL receptor expression, lipoprotein lipase activity, and triglyceride-rich lipoprotein clearance [37]. Therefore, even mild thyroid-axis alterations may worsen lipid handling. In parallel, autoimmune inflammation may contribute to oxidative stress, adipokine disturbance, and low-grade inflammatory changes that promote an atherogenic lipid phenotype [39].

From a clinical perspective, SIRI, PNI, and AIP may serve as practical adjunctive markers for characterizing the systemic profile of HT. Their associations with thyroid functional status suggest that inflammatory and atherogenic alterations may be present even in EH, whereas lower PNI may reflect immunonutritional alterations associated with SCH or OH. Their combined interpretation should account for shared components among the evaluated indices: lymphocyte count contributes to several inflammatory indices and to PNI and CONUT; HDL-C contributes to AIP and the Castelli indices; and CONUT shares albumin with PNI and total cholesterol with Castelli I. Consequently, some inter-index correlations may partly reflect mathematical coupling in addition to biological relationships. Although Elastic Net–penalized regression was used to accommodate correlated candidate variables during selection, it does not eliminate mathematical coupling in the correlation analyses. Because all three indices are derived from routine laboratory tests, they may help identify patients who warrant closer metabolic and nutritional assessment.

### Limitations

This study has several limitations. First, the retrospective single-center design limits causal inference and may have introduced selection bias. The cross-sectional comparisons assessed associations with thyroid functional status at baseline in different individuals and could not establish within-patient changes, temporal relationships, or individual disease trajectories. Second, although strict exclusion criteria reduced confounding from comorbid diseases, smoking, alcohol use, medications, and supplements, they also produced a selected cohort that may not reflect the broader HT population encountered in routine practice. Third, despite propensity score matching, residual imbalance in sex distribution remained between the HT and control groups, and this may have influenced inflammatory and lipid-related indices. Fourth, the subgroup sample sizes, particularly for OH, were modest, which may affect the stability of multivariable regression and ROC estimates. Fifth, formal nutritional assessment, body composition analysis, dietary intake, physical activity, iodine status, vitamin D status, thyroid volume, detailed ultrasonographic severity, cytokine levels, oxidative stress markers, and lipid subfractions were not available. Accordingly, the observed differences in PNI and CONUT cannot establish clinically defined nutritional impairment. Sixth, potential interassay or interplatform variability during the 2015–2025 study period could not be excluded and may have influenced laboratory measurements and derived indices. Finally, all cutoff values were derived from the present cohort and were not externally validated. Prospective multicenter studies with longitudinal follow-up are needed to determine whether SIRI, PNI, and AIP predict subsequent changes in thyroid function and whether longitudinal changes in these indices are associated with treatment response and long-term cardiometabolic outcomes.

## 5. Conclusions

Inflammatory, immunonutritional, and atherogenic profiles differed across thyroid functional groups in patients with HT. Among routinely available indices, SIRI and AIP were independently associated with EH, suggesting that inflammatory and lipid-related alterations may be present without overt thyroid dysfunction. In the SCH and OH groups, PNI was also identified as an independent marker, indicating that immunonutritional alterations are more pronounced in the hypothyroid groups. These findings support the potential value of evaluating SIRI, PNI, and AIP together as an adjunctive panel for characterizing the systemic burden of HT.

## Figures and Tables

**Table 1:** Comparison of inflammatory, immunonutritional, and atherogenic lipid indices between the Hashimoto’s thyroiditis and control groups.

Variables	All Population	Control	HT	*p*
	*n* = 222	*n* = 74	*n* = 148	
Inflammatory indices				
NLR	1.7 (1.3–2.3)	1.3 (1.0–1.7)	2.0 (1.6–2.6)	<0.001 *
PLR	114.6 (95.2–142.8)	101.3 (89.6–132.8)	120.7 (97.8–144.7)	0.016 *
LMR	4.8 (3.5–6.1)	5.6 (4.7–7.2)	4.1 (3.3–5.4)	<0.001 *
SII	443.8 (307.1–573.5)	306.6 (225.3–398.3)	516.7 (375.9–665.2)	<0.001 *
SIRI	0.8 (0.6–1.1)	0.5 (0.4–0.7)	1.0 (0.8–1.3)	<0.001 *
CAR	0.7 (0.4–1.0)	0.5 (0.3–0.8)	0.8 (0.4–1.3)	<0.001 *
Immunonutritional indices				
PNI	49.4 ± 8.3	56.0 ± 3.2	46.1 ± 8.0	<0.001 *
CONUT score	1.0 (0.0–2.0)	0.0 (0.0–1.0)	1.0 (0.0–3.0)	<0.001 *
Atherogenic lipid indices				
AIP	0.3 (0.2–0.6)	0.1 (0.0–0.3)	0.5 (0.3–0.6)	<0.001 *
Castelli I index	3.8 ± 1.1	3.3 ± 0.9	4.1 ± 1.0	<0.001 *
Castelli II index	2.3 ± 0.8	2.0 ± 0.7	2.5 ± 0.8	<0.001 *

Data are presented as mean ± standard deviation, median (interquartile range), or number (%), as appropriate. * *p* < 0.05 indicates statistical significance. Abbreviations: AIP, atherogenic index of plasma; CAR, C-reactive protein-to-albumin ratio; CONUT, Controlling Nutritional Status; HT, Hashimoto’s thyroiditis; LMR, lymphocyte-to-monocyte ratio; NLR, neutrophil-to-lymphocyte ratio; PLR, platelet-to-lymphocyte ratio; PNI, prognostic nutritional index; SII, systemic immune-inflammation index; SIRI, systemic inflammation response index.

**Table 2:** Inflammatory, immunonutritional, and atherogenic lipid indices according to thyroid functional status.

Variables	Control	EH	SCH	OH	*p*
	*n* = 74	*n* = 62	*n* = 47	*n* = 39	
Inflammatory indices					
NLR	1.2 (1.0–1.7)	1.7 (1.4–2.1)	2.1 (1.8–2.9)	2.6 (1.9–3.4)	<0.001 *
PLR	101.3 (89.6–132.8)	118.3 (95.2–145.3)	127.4 (103.2–140.3)	136.4 (112.0–159.9)	0.035 *
LMR	5.6 (4.7–7.2)	4.7 (3.8–6.2)	4.3 (3.2–5.4)	3.3 (2.8–4.1)	<0.001
SII	306.6 (225.3–398.3)	401.0 (322.1–508.6)	445.2 (346.1–533.5)	504.0 (405.9–645.0)	<0.001
SIRI	0.5 (0.4–0.7)	0.8 (0.6–1.0)	1.0 (0.9–1.4)	1.4 (1.2–1.7)	<0.001 *
CAR	0.5 (0.3–0.8)	0.5 (0.3–0.7)	1.0 (0.6–1.3)	1.3 (0.8–2.3)	<0.001 *
Immunonutritional indices					
PNI	56.0 ± 3.2	50.2 ± 6.0	46.8 ± 5.5	40.6 ± 7.0	<0.001
CONUT score	0.0 (0.0–1.0)	1.0 (0.0–2.0)	1.0 (0.0–2.0)	3.0 (1.0–5.0)	<0.001 *
Atherogenic lipid indices					
AIP	0.1 (0.0–0.3)	0.3 (0.2–0.5)	0.5 (0.4–0.6)	0.8 (0.5–0.9)	<0.001
Castelli I index	3.3 ± 0.9	3.5 ± 0.7	4.3 ± 1.0	4.9 ± 1.1	<0.001 *
Castelli II index	2.0 ± 0.7	2.2 ± 0.6	2.6 ± 0.8	2.9 ± 0.9	<0.001 *

Data are presented as mean ± standard deviation, median (interquartile range), or number (%), as appropriate. Bold values indicate statistically significant differences between groups. Abbreviations: see Table 1; EH, euthyroid Hashimoto thyroiditis; SCH, subclinical hypothyroidism; OH, overt hypothyroidism. * *p* < 0.05 indicates statistical significance.

**Table 3:** Independent predictors of thyroid functional status.

Variables	Crude Regression		Multivariable Regression		
	OR (95% CI)	*p*	OR (95% CI)	*p*	VIF
EH vs. controls					
SII	1.82 (1.39–2.38)	<0.001	1.30 (0.78–2.16)	0.319	2.04
SIRI	2.08 (1.63–2.66)	<0.001	2.15 (1.42–3.27)	<0.001	2.27
PNI	0.83 (0.74–0.93)	0.004	0.88 (0.77–1.01)	0.082	1.82
CONUT score	1.60 (1.16–2.21)	0.004	1.35 (0.75–2.45)	0.318	1.57
AIP	2.14 (1.62–2.83)	<0.001	2.47 (1.62–3.77)	<0.001	1.20
			Nagelkerke R^2^ = 0.641		
SCH vs. EH					
SIRI	1.58 (1.29–1.93)	<0.001	1.75 (1.30–2.35)	<0.001	1.18
CAR	1.12 (1.01–1.25)	<0.001	1.16 (0.95–1.37)	0.091	1.19
PNI	0.91 (0.87–0.95)	<0.001	0.92 (0.86–0.98)	<0.001	1.23
AIP	2.07 (1.54–2.78)	<0.001	2.32 (1.46–3.70)	<0.001	1.54
Castelli II index	2.15 (1.28–3.63)	0.004	1.13 (0.51–2.49)	0.768	1.42
			Nagelkerke R^2^ = 0.664		
OH vs. SCH					
SII	1.09 (1.01–1.18)	0.033	0.95 (0.51–1.77)	0.865	1.47
SIRI	1.29 (1.12–1.49)	<0.001	1.33 (1.03–1.72)	0.005 *	1.75
CAR	1.12 (1.05–1.19)	0.001	1.11 (0.93–1.32)	0.254	1.42
PNI	0.79 (0.72–0.87)	<0.001	0.82 (0.70–0.96)	0.007 *	3.12
CONUT score	1.90 (1.44–2.52)	<0.001	2.02 (0.98–4.16)	0.078	3.04
AIP	1.84 (1.35–2.51)	<0.001	2.15 (1.44–3.21)	0.001	1.23
Castelli II index	1.37 (1.06–1.77)	0.184	1.89 (0.95–3.76)	0.368	1.44
			Nagelkerke R^2^ = 0.640		

All models were adjusted for age, gender, and BMI. * *p* < 0.05 indicates statistical significance. Abbreviations: see Table 1; EH, euthyroid Hashimoto’s thyroiditis; SCH, subclinical hypothyroidism; OH, overt hypothyroidism; BMI, body mass index; CI, confidence interval; OR, odds ratio; VIF, variance inflation factor.

## Data Availability

The data that support the findings of this study are available on request from the corresponding author.

## Supplementary Materials

The following supporting information can be downloaded at: https://www.mdpi.com/article/10.3390/jcm15218607/s1, Table S1: Overview of demographic characteristics, thyroid function status, laboratory parameters, and calculated indices evaluated in the study population; Table S2: Formulas of composite inflammatory, immunonutritional, and atherogenic indices. Table S3: Demographic and clinical characteristics of the study population. Supplementary Table S4. Spearman correlation matrix with Benjamini–Hochberg FDR-adjusted q values. Table S5: Demographic and clinical characteristics according to HT subgroup. Table S6. Diagnostic performance of inflammatory, nutritional, and atherogenic indices for thyroid functional status.

## Abbreviations

The following abbreviations are used in this manuscript:

^18^F-FDG	Fluorine-18 fluorodeoxyglucose
AIP	Atherogenic index of plasma
anti-Tg	Anti-thyroglobulin antibody
anti-TPO	Anti-thyroid peroxidase antibody
AUC	Area under the receiver operating characteristic curve
BMI	Body mass index
CAR	C-reactive protein-to-albumin ratio
CI	Confidence interval
CONUT	Controlling Nutritional Status
CRP	C-reactive protein
CV	Cross-validation
EH	Euthyroid Hashimoto’s thyroiditis
FDR	False discovery rate
fT3	Free triiodothyronine
fT4	Free thyroxine
HDL-C	High-density lipoprotein cholesterol
HT	Hashimoto’s thyroiditis
IFN-γ	Interferon-γ
IL-1	Interleukin-1
IL-23	Interleukin-23
IL-6	Interleukin-6
LDL-C	Low-density lipoprotein cholesterol
LMR	Lymphocyte-to-monocyte ratio
NLR	Neutrophil-to-lymphocyte ratio
OH	Overt hypothyroidism
OR	Odds ratio
PLR	Platelet-to-lymphocyte ratio
PNI	Prognostic nutritional index
ROC	Receiver operating characteristic
SCH	Subclinical hypothyroidism
SII	Systemic immune-inflammation index
SIRI	Systemic inflammation response index
TNF-α	Tumor necrosis factor-α
TSH	Thyroid-stimulating hormone
VIF	Variance inflation factor
