# Supplementary material for: Evaluation of Inflammatory, Immunonutritional, and Atherogenic Profiles in Patients with Hashimoto’s Thyroiditis
Source: J Clin Med. 2026 Nov 5;15(21):8607. doi: 10.3390/jcm15218607 (PMC13764037; doi:10.3390/jcm15218607)
Supplement: Supplementary file 1 [file jcm-15-08607-s001.zip › jcm-4546472-supplementary.pdf]

Supplementary Table S1. Overview of demographic characteristics, thyroid function status, laboratory parameters, and calculated indices evaluated in the study population.

**Class**	**Variables**
Demographic	Age<br>Gender<br>BMI<br>Blood pressure
Thyroid function	Anti-thyroglobulin (anti-Tg)<br>Anti-thyroid peroxidase (anti-TPO)<br>Thyroid-stimulating hormone (TSH)<br>free thyroxine (fT4)
HT subgroup	Euthyroid (EH)<br>Subclinical hypothyroidism (SCH)<br>Overt hypothyroidism (OH)
CBC parameters	Hemoglobin<br>Leukocytes<br>Neutrophils<br>Lymphocytes<br>Monocytes<br>Platelets
Acute-phase and biochemical	C-reactive protein (CRP)<br>Albumin<br>Alanine aminotransferase (ALT)<br>Aspartate aminotransferase (AST)<br>Urea<br>Creatinine
Inflammatory indices	Neutrophil-to-lymphocyte ratio (NLR)<br>Platelet-to-lymphocyte ratio (PLR)<br>Lymphocyte-to-monocyte ratio (LMR)<br>Systemic immune-inflammation index (SII)<br>Systemic inflammation response index (SIRI)<br>CRP to albumin ratio (CAR)
Lipid profile	Total cholesterol<br>High-density lipoprotein (HDL) cholesterol<br>Low-density lipoprotein (LDL) cholesterol<br>Triglycerides
Immunonutritional indices	Prognostic nutritional index (PNI)<br>Controlling Nutritional Status (CONUT) score
Atherogenic lipid indices	Atherogenic index of plasma (AIP)<br>Castelli Risk Index I<br>Castelli Risk Index II

All laboratory values used in the study were obtained at the time of initial diagnosis.

Supplementary Table S2. Formulas of composite inflammatory, immunonutritional, and atherogenic indices**.**

Index	Formula
NLR	Neutrophils / Lymphocytes ratio
PLR	Platelets / Lymphocytes ratio
LMR	Lymphocytes / Monocytes ratio
SII	(Platelets × Neutrophils) / Lymphocytes ratio
SIRI	(Neutrophils × Monocytes) / Lymphocytes ratio
CAR	CRP / Albumin ratio
PNI	10 × Albumin + 5 × Lymphocytes
CONUT score	Albumin score + lymphocyte score + total cholesterol score
AIP	$\log_{10}$(Triglycerides / HDL-C)
Castelli I index	Total cholesterol / HDL-C ratio
Castelli II index	LDL-C / HDL-C ratio

PNI was calculated according to the Onodera formula using serum albumin and total lymphocyte count. AIP was calculated according to the definition proposed by Dobiášová and Frohlich as the base-10 logarithm of the molar triglyceride-to-HDL-C ratio. Accordingly, triglyceride and HDL-C concentrations reported in mg/dL were conver-ted to mmol/L before calculation of AIP.

Supplementary Table S3. Demographic and clinical characteristics of the study population.

Variables	All population n = 222	Control n = 74	HT n = 148	p
Age, years	43.1 ± 14.3	41.5 ± 14.0	43.9 ± 14.4	0.239
Female sex, n (%)	165 (74.3)	53 (71.6)	112 (75.7)	0.625
BMI, kg/m^2	27.5 ± 5.1	26.5 ± 4.4	28.3 ± 5.4	0.033*
SBP, mmHg	123.8 ± 13.9	122.1 ± 12.7	124.7 ± 14.4	0.173
DBP, mmHg	72.2 ± 11.0	72.1 ± 8.8	72.3 ± 12.0	0.904
Laboratory findings				
TSH, mIU/L	3.0 (1.6–6.4)	1.6 (1.1–2.1)	6.0 (2.8–7.5)	<0.001*
fT4, ng/dL	0.8 ± 0.2	1.0 ± 0.1	0.8 ± 0.2	<0.001*
Anti-TPO positivity, n (%)	127 (57.2)	2 (2.7)	125 (84.5)	<0.001*
Anti-Tg positivity, n (%)	94 (42.3)	2 (2.7)	92 (62.2)	<0.001*
Hemoglobin, g/dL	12.9 ± 1.6	13.1 ± 1.8	12.8 ± 1.5	0.220
Leukocytes, ×10^3/μL	7.1 ± 2.0	6.1 ± 1.4	7.5 ± 2.1	<0.001*
Neutrophils, ×10^3/μL	4.1 ± 1.6	3.0 ± 1.0	4.6 ± 1.6	<0.001*
Lymphocytes, ×10^3/μL	2.2 (1.8–2.7)	2.3 (2.0–2.7)	2.1 (1.7–2.7)	0.044*
Monocytes, ×10^3/μL	0.5 ± 0.1	0.4 ± 0.1	0.5 ± 0.1	<0.001*
Platelets, ×10^3/μL	252.2 ± 64.5	249.7 ± 62.4	253.5 ± 65.6	0.677
CRP, mg/L	2.5 (1.6–4.2)	1.8 (1.2–2.7)	2.9 (1.6–4.5)	0.125
Albumin, g/dL	3.8 ± 0.7	4.4 ± 0.2	3.5 ± 0.7	<0.001*
ALT, IU/L	17.0 (12.0–25.0)	17.0 (13.0–23.0)	16.0 (12.0–26.0)	0.730
AST, IU/L	20.0 (16.0–23.0)	20.0 (16.0–23.0)	20.0 (16.0–23.0)	0.974
Urea, mg/dL	25.8 ± 12.4	25.5 ± 8.9	26.0 ± 13.9	0.706
Creatinine, mg/dL	0.7 ± 0.2	0.7 ± 0.2	0.7 ± 0.2	0.377
Total cholesterol, mg/dL	198.1 ± 46.5	185.1 ± 42.6	204.5 ± 47.1	0.003*
HDL-C, mg/dL	53.2 ± 11.4	58.3 ± 12.5	50.6 ± 10.0	<0.001*
LDL-C, mg/dL	119.0 ± 39.5	110.9 ± 38.2	123.1 ± 39.6	0.030*
Triglycerides, mg/dL	114.0 (84.2–178.0)	80.0 (62.0–98.0)	149.0 (107.2–199.0)	<0.001*

Data are presented as mean ± standard deviation, median (interquartile range), or number (%), as appropriate. *p<0.05 indicates statistical significance. Abbreviations: AIP, atherogenic index of plasma; BMI, body mass index; CAR, C-reactive protein-to-albumin ratio; CONUT, Controlling Nutritional Status; CRP, C-reactive protein; DBP, diastolic blood pressure; fT4, free thyroxine; HDL-C, high-density lipoprotein cholesterol; HT, Hashimoto's thyroiditis; LDL-C, low-density lipoprotein cholesterol; LMR, lymphocyte-to-monocyte ratio; NLR, neutrophil-to-lymphocyte ratio; PLR, platelet-to-lymphocyte ratio; PNI, prognostic nutritional index; SBP, systolic blood pressure; SII, systemic immune-inflammation index; SIRI, systemic inflammation response index; TSH, thyroid-stimulating hormone; EH, euthyroid Hashimoto thyroiditis; SCH, subclinical hypothyroidism; OH, overt hypothyroidism.

Supplementary Table S4. Spearman correlation matrix with Benjamini–Hochberg FDR-adjusted q values.

	BMI	TSH	fT4	anti-TPO	anti-Tg	NLR	PLR	LMR	SII	SIRI	CAR	PNI	CONUT	AIP	Castelli I
TSH	0.071 (0.324)	—													
fT4	-0.023 (0.742)	-0.516 (<0.001)	—												
anti-TPO	0.157 (0.023)	0.485 (<0.001)	-0.321 (<0.001)	—											
anti-Tg	0.024 (0.732)	0.357 (<0.001)	-0.190 (0.006)	0.354 (<0.001)	—										
NLR	0.067 (0.351)	0.502 (<0.001)	-0.442 (<0.001)	0.394 (<0.001)	0.411 (<0.001)	—									
PLR	-0.040 (0.583)	0.122 (0.083)	-0.074 (0.303)	0.087 (0.224)	0.091 (0.203)	0.399 (<0.001)	—								
LMR	0.158 (0.023)	-0.392 (<0.001)	0.310 (<0.001)	-0.215 (0.002)	-0.195 (0.005)	-0.408 (<0.001)	-0.526 (<0.001)	—							
SII	0.144 (0.038)	0.425 (<0.001)	-0.333 (<0.001)	0.418 (<0.001)	0.346 (<0.001)	0.834 (<0.001)	0.594 (<0.001)	-0.212 (0.002)	—						
SIRI	0.058 (0.418)	0.668 (<0.001)	-0.515 (<0.001)	0.529 (<0.001)	0.464 (<0.001)	0.886 (<0.001)	0.322 (<0.001)	-0.601 (<0.001)	0.738 (<0.001)	—					
CAR	0.142 (0.042)	0.432 (<0.001)	-0.190 (0.006)	0.167 (0.016)	0.171 (0.014)	0.300 (<0.001)	0.174 (0.012)	-0.214 (0.002)	0.317 (<0.001)	0.359 (<0.001)	—				
PNI	0.054 (0.449)	-0.548 (<0.001)	0.452 (<0.001)	-0.455 (<0.001)	-0.360 (<0.001)	-0.550 (<0.001)	-0.398 (<0.001)	0.651 (<0.001)	-0.443 (<0.001)	-0.663 (<0.001)	-0.397 (<0.001)	—			
CONUT	-0.033 (0.639)	0.348 (<0.001)	-0.381 (<0.001)	0.194 (0.005)	0.157 (0.023)	0.294 (<0.001)	0.196 (0.005)	-0.365 (<0.001)	0.240 (<0.001)	0.387 (<0.001)	0.268 (<0.001)	-0.637 (<0.001)	—		
AIP	0.042 (0.563)	0.649 (<0.001)	-0.406 (<0.001)	0.516 (<0.001)	0.364 (<0.001)	0.477 (<0.001)	0.171 (0.014)	-0.378 (<0.001)	0.411 (<0.001)	0.614 (<0.001)	0.319 (<0.001)	-0.533 (<0.001)	0.214 (0.002)	—	
Castelli I	0.071 (0.325)	0.487 (<0.001)	-0.305 (<0.001)	0.345 (<0.001)	0.262 (<0.001)	0.307 (<0.001)	0.012 (0.855)	-0.221 (0.001)	0.230 (<0.001)	0.394 (<0.001)	0.297 (<0.001)	-0.375 (<0.001)	-0.036 (0.623)	0.725 (<0.001)	—
Castelli II	0.086 (0.231)	0.366 (<0.001)	-0.218 (0.002)	0.278 (<0.001)	0.234 (<0.001)	0.234 (<0.001)	-0.033 (0.639)	-0.137 (0.049)	0.164 (0.018)	0.280 (<0.001)	0.215 (0.002)	-0.281 (<0.001)	-0.114 (0.106)	0.549 (<0.001)	0.942 (<0.001)

Data are shown as Spearman's correlation coefficient (ϱ) with Benjamini–Hochberg false discovery rate–adjusted q values in parentheses.

Supplementary Table S5. Demographic and clinical characteristics according to HT subgroup.

Variables	Control n = 74	EH n = 62	SCH n = 47	OH n = 39	p
Age, years	41.5 ± 14.0	43.8 ± 12.7	44.1 ± 15.6	43.8 ± 15.9	0.707
Female sex, n (%)	53 (71.6)	49 (79.0)	28 (71.8)	35 (74.5)	0.762
BMI, kg/m²	26.5 ± 4.4	27.2 ± 5.6	28.0 ± 5.1	**30.3 ± 5.2**	0.038*
SBP, mmHg	122.1 ± 12.7	124.5 ± 12.9	122.4 ± 12.5	127.9 ± 18.2	0.166
DBP, mmHg	72.1 ± 8.8	72.2 ± 12.2	71.3 ± 11.4	73.6 ± 12.6	0.819
Laboratory findings					
TSH, mIU/L	**1.6 (1.1–2.1)**	**2.4 (1.3–3.7)**	**6.2 (6.0–7.5)**	**9.3 (7.2–12.9)**	<0.001*
fT4, ng/dL	**1.0 ± 0.1**	**0.9 ± 0.1**	**0.9 ± 0.1**	**0.5 ± 0.1**	<0.001
Anti-TPO positivity, n (%)	**2 (2.7)**	**53 (85.5)**	**37 (78.7)**	**35 (89.7)**	<0.001*
Anti-Tg positivity, n (%)	**2 (2.7)**	**37 (59.7)**	**34 (72.3)**	**21 (53.8)**	<0.001*
Hemoglobin, g/dL	13.1 ± 1.8	12.9 ± 1.7	12.9 ± 1.4	12.6 ± 1.5	0.517
Leukocytes, ×10³/μL	**6.1 ± 1.4**	**7.1 ± 2.0**	**7.7 ± 2.1**	**8.0 ± 2.3**	<0.001*
Neutrophils, ×10³/μL	**3.0 ± 1.0**	**4.0 ± 1.3**	**4.9 ± 1.5**	**5.1 ± 1.8**	<0.001*
Lymphocytes, ×10³/μL	2.3 (2.0–2.7)	2.2 (1.8–2.6)	**2.0 (1.6–2.3)**	**2.0 (1.6–2.2)**	0.038*
Monocytes, ×10³/μL	**0.4 ± 0.1**	**0.5 ± 0.1**	**0.6 ± 0.1**	**0.7± 0.1**	<0.001
Platelets, ×10³/μL	249.7 ± 62.4	255.6 ± 58.8	257.5 ± 73.2	265.4 ± 67.2	0.395
CRP, mg/L	**1.8 (1.2–2.7)**	**2.4 (1.4–3.8)**	3.6 (2.7–4.5)	3.8 (2.1–5.4)	<0.001*
Albumin, g/dL	**4.4 ± 0.2**	3.9 ± 0.5	3.7 ± 0.4	**2.7 ± 0.6**	<0.001
ALT, IU/L	17.0 (13.0–23.0)	16.5 (11.2–24.0)	17.0 (12.0–26.0)	16.0 (12.0–21.5)	0.810
AST, IU/L	20.0 (16.0–23.0)	20.0 (15.2–24.0)	19.0 (15.5–23.0)	20.0 (16.0–22.0)	0.948
Urea, mg/dL	25.5 ± 8.9	26.1 ± 8.2	25.3 ± 7.0	29.2 ± 23.8	0.287
Creatinine, mg/dL	0.7 ± 0.2	0.8 ± 0.2	0.7 ± 0.2	0.7 ± 0.2	0.281
Total cholesterol, mg/dL	185.1 ± 42.6	190.0 ± 42.7	204.2 ± 44.0	**228.2 ± 48.8**	<0.001*
HDL-C, mg/dL	58.3 ± 12.5	56.4 ± 8.5	**51.3 ± 11.0**	**47.9 ± 10.1**	<0.001*
LDL-C, mg/dL	110.9 ± 38.2	113.9 ± 34.3	**124.2 ± 39.0**	**136.5 ± 44.8**	0.005*
Triglycerides, mg/dL	**80.0 (62.0–98.0)**	**106.5 (87.0–142.8)**	**149.0 (125.0–189.0)**	**244.0 (183.0–315.5)**	<0.001*

Data are presented as mean ± standard deviation, median (interquartile range), or number (%), as appropriate. *p<0.05 indicates statistical significance. Bold values indicate statistically significant differences between groups. Abbreviations: see Supplementary Table S3.

Supplementary Table S6. Diagnostic performance of inflammatory, nutritional, and atherogenic indices for thyroid functional status.

Index / comparison	AUC ± SE	Sensitivity (%) (95% CI)	Specificity (%) (95% CI)	Cut-off value	p
EH vs control					
NLR	0.690 ± 0.04	73.5 (64.6–77.5)	64.1 (42.8–64.9)	≥1.21	<0.001
PLR	0.657 ± 0.05	61.3 (48.8–72.4)	62.2 (50.8–72.4)	≥107.6	0.047
LMR	0.655 ± 0.05	51.6 (39.4–63.6)	70.7 (59.8–80.0)	≤4.69	0.001
SII	0.689 ± 0.04	61.3 (48.8–72.4)	70.0 (59.8–79.0)	≥399.3	<0.001
SIRI	0.734 ± 0.04	76.4 (70.4–85.7)	73.3 (64.1–82.5)	≥0.54	<0.001
CAR	0.534 ± 0.05	69.0 (57.4–77.3)	37.8 (27.6–49.2)	≤0.79	0.490
PNI	0.683 ± 0.04	69.7 (47.3–71.0)	65.4 (58.9–75.6)	≤51.5	<0.001
CONUT score	0.597 ± 0.05	32.3 (22.0–44.6)	76.5 (66.9–82.5)	≥2.00	0.048
AIP	0.743 ± 0.04	77.1 (66.6–83.3)	75.5 (70.1–80.9)	≥0.19	<0.001
Castelli I index	0.627 ± 0.05	68.7 (58.5–74.4)	55.9 (45.1–67.2)	≥2.91	0.008
Castelli II index	0.603 ± 0.05	71.9 (62.5–86.5)	57.8 (47.6–69.2)	≥1.37	0.035
SCH vs EH					
NLR	0.697 ± 0.05	68.7 (55.1–78.0)	65.3 (53.8–77.4)	≥1.79	<0.001
PLR	0.543 ± 0.06	66.6 (52.8–76.4)	37.1 (26.2–49.5)	≥103.2	0.448
LMR	0.683 ± 0.06	55.5 (45.3–69.5)	75.2 (66.7–88.3)	≤3.12	0.033
SII	0.667 ± 0.05	48.9 (35.3–62.8)	77.4 (65.6–86.0)	≥566.2	0.002
SIRI	0.726 ± 0.05	73.6 (62.8–77.8)	68.4 (56.4–80.6)	≥0.80	<0.001
CAR	0.693 ± 0.05	61.7 (47.4–74.2)	73.9 (62.8–81.0)	≥0.95	0.035
PNI	0.716 ± 0.05	70.1 (55.8–81.6)	70.5 (58.1–82.1)	≤50.0	<0.001
CONUT score	0.507 ± 0.06	63.8 (49.5–76.0)	41.9 (30.5–54.3)	≥1.00	0.900
AIP	0.754 ± 0.04	78.1 (63.8–89.6)	80.5 (69.7–87.2)	≥0.49	<0.001
Castelli I index	0.656 ± 0.05	48.3 (35.8–62.6)	73.5 (64.6–77.5)	≥4.65	<0.001
Castelli II index	0.644 ± 0.05	50.4 (37.6–64.7)	73.5 (64.6–77.5)	≥3.10	0.008
OH vs SCH					
NLR	0.689 ± 0.06	68.1 (52.4–81.3)	68.7 (54.4–81.2)	≥2.27	0.029
PLR	0.640 ± 0.05	53.6 (39.3–69.0)	68.3 (54.2–79.1)	≥136.2	0.048
LMR	0.690 ± 0.06	71.8 (56.2–83.5)	66.0 (51.7–77.8)	≤3.85	0.001
SII	0.686 ± 0.05	70.9 (55.7–81.4)	66.2 (54.0–80.5)	≥514.0	0.037
SIRI	0.756 ± 0.06	75.8 (61.2–88.5)	74.2 (60.0–85.3)	≥1.25	<0.001
CAR	0.671 ± 0.06	61.0 (47.1–76.6)	75.7 (65.8–78.8)	≥1.76	0.004
PNI	0.730 ± 0.04	73.7 (60.4–81.9)	72.3 (58.2–83.1)	≤46.5	<0.001
CONUT score	0.718 ± 0.05	70.7 (55.0–84.4)	70.7 (60.8–80.8)	≥4.00	<0.001
AIP	0.732 ± 0.06	76.2 (61.6–91.4)	78.6 (70.4–86.2)	≥0.71	<0.001
Castelli I index	0.661 ± 0.06	72.5 (59.3–87.3)	60.4 (47.6–74.7)	≥3.75	0.007
Castelli II index	0.583 ± 0.06	72.3 (59.7–77.3)	47.7 (36.9–61.8)	≥1.91	0.006

Abbreviations: see Supplementary Table S3; AUC, area under the receiver operating characteristic curve; CI, confidence interval; SE, standard error.
